# Analysis of Long Noncoding RNA ZNF667-AS1 as a Potential Biomarker for Diagnosis and Prognosis of Glioma Patients

**DOI:** 10.1155/2020/8895968

**Published:** 2020-11-16

**Authors:** Qin Yuan, Chao Gao, Xiao-dong Lai, Liang-yi Chen, Tian-bao Lai

**Affiliations:** ^1^Department of Oncology and Hematology, Hubei Provincial Hospital of Integrated Chinese and Western Medicine, Wuhan, 430015 Hubei, China; ^2^Department of Oncology, First People's Hospital of Zaoyang, Zaoyang, 441200 Hubei, China; ^3^Fuzhou Medical College of Nanchang University, Fuzhou, 344000 Nanchang, China; ^4^Department of Neurology, Zhongshan Hospital Affiliated to Xiamen University, Xiamen, 361004 Fujian, China

## Abstract

**Objective:**

Long noncoding RNAs (lncRNAs) have been strongly associated with various types of cancer. The present study aimed at exploring the diagnostic and prognostic value of lncRNA Zinc finger protein 667-antisense RNA 1 (ZNF667-AS1) in glioma patients. *Patients and Methods*. The expressions of ZNF667-AS1 were detected in 155 glioma tissues and matched normal brain tissue samples by qRT-PCR. The receiver operating characteristic (ROC) curve was performed to estimate the diagnostic value of ZNF667-AS1. The association between the ZNF667-AS1 expression and clinicopathological characteristics was analyzed by the chi-square test. The Kaplan-Meier method was performed to determine the influence of the ZNF667-AS1 expression on the overall survival and disease-free survival of glioma patients. The Cox regression analysis was used to evaluate the effect of independent prognostic factors on survival outcome. Cell proliferation was measured by the respective cell counting Kit-8 (CCK-8) assays.

**Results:**

We observed that ZNF667-AS1 was significantly upregulated in glioma tissues compared to normal tissue samples (*p* < 0.01). Higher levels of ZNF667-AS1 were positively associated with the WHO grade (*p* = 0.018) and KPS score (*p* = 0.008). ROC assays revealed that the high ZNF667-AS1 expression had an AUC value of 0.8541 (95% CI: 0.8148 to 0.8934) for glioma. Survival data revealed that glioma patients in the high ZNF667-AS1 expression group had significantly shorter 5-year overall survival (*p* = 0.0026) and disease-free survival (*p* = 0.0005) time than those in the low ZNF667-AS1 expression group. Moreover, multivariate analyses confirmed that the ZNF667-AS1 expression was an independent predictor of the overall survival and disease-free survival for glioma patients. Functionally, we found that knockdown of ZNF667-AS1 suppressed the proliferation of glioma cells.

**Conclusions:**

Our results suggest that ZNF667-AS1 could be used as a potential diagnostic and prognostic biomarker in glioma.

## 1. Introduction

Human gliomas refer to the commonest malignant tumors of the core nervous mechanism, achieving ~6/100,000 incidence each year globally [1]. Malignant glioma exhibits aggressiveness, high infiltration, and resistance to normal therapies [2]. It is noteworthy that glioblastoma multiform (GBM), a particular glioma kind, exhibits the highest aggressiveness for the rapid growing process and frequent spread to close brain-related tissues [3]. Though the overall treating condition of brain gliomas continues to progress, such malignancy outcome is not optimized significantly [4, 5]. Thus, emerging molecular markers should be developed for improving gliomas classifying process, patient prognosis, and treating plans.

Over the past few years, according to research with various overall genome methods, e.g., the ENCODE program, most mammalian genome receives transcription, whereas only 1.2% of the mentioned transcripts suggest the genes coding protein [6, 7]. Long noncoding RNAs (lncRNAs) refer to a sequence of transcripts with over 200 nt exhibiting slight or no ability to the code protein and critically impact a wide range of cellular processes, e.g., splicing, X chromosome inactivation, epigenetic controls, and gene transcription regulation [8, 9]. Numerous lncRNAs suggested aberrant expression in various cancer types, and several lncRNAs with dysregulation become oncogenes or tumor-suppressing elements under special cases [10, 11]. For instance, lncRNA NFIA-AS2 was shown to be highly expressed in glioma and promoted the proliferation and migration of glioma cells via modulating the miRNA-655-3p/ZFX axis [12]. LncRNA RNCR3 was shown to promote the proliferation and metastasis of glioma cells via regulating the Akt/GSK-3*β* pathway [13]. In addition, more and more lncRNAs were identified to be potential diagnostic and prognostic biomarkers for various tumor patients, including glioma [14–17]. Although several studies have investigated lncRNAs in glioma, their completely biological roles and clinical significance are still obscure.

LncRNA Zinc finger protein 667-antisense RNA 1 (ZNF667-AS1) in chromosome 19q13.43 of human was recently identified as tumor-related lncRNA. Previously, several studies have reported that ZNF667-AS1 was abnormally expressed in several tumor specimens and cells by the use of RT-PCR and high-throughput sequencing [18–20]. In addition, its tumor-related functions acting as tumor promotors or antioncogenes have been reported in nasopharyngeal carcinoma, esophageal squamous cell carcinoma, and cervical cancer [21–23]. However, whether ZNF667-AS1 also displayed an abnormal expression in glioma had not been investigated. Here, its expressing pattern and clinical significance in glioma patients are to be primarily explored.

## 2. Patients and Methods

### 2.1. Patient Data and Tissue Samples

Patients with glioma admitted to the oncology from June 2012 to August 2015 were drawn from the hospital tumor registry. The 155 enrolled patients were aged from 29 to 65 years, with a median age of 42 years. Histological diagnoses were made according to the 2007 WHO classification. 141 glioma patients completed follow-up, and the rate of loss to follow-up was 9%. All the patients did not receive prior anticancer treatment.

**Figure 1:**
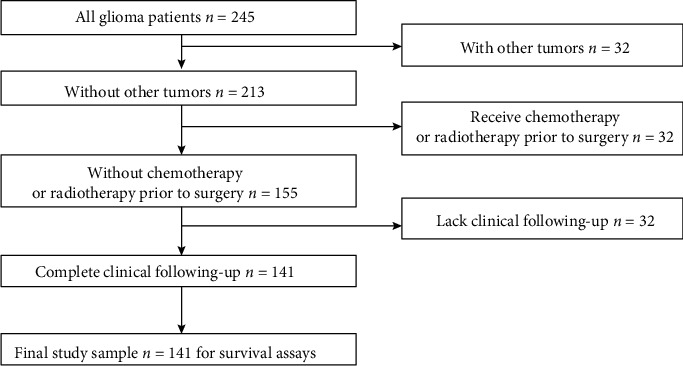
Flow chart in the selection of study patients.

The flow chart in the section of study patients was shown in Figure 1. This study proved overall samples by pathology-related tests and incubated them in liquid nitrogen to achieve the subsequent overall RNA extracting process. The clinical and pathological data for the patients are reported in Table 1. The present study was ethically approved by the Research Ethics Committee of the Hubei Provincial Hospital of Integrated Chinese and Western Medicine (number: 20180056), and written informed consent was acquired from patient.

### 2.2. Cell Culture and siRNA Transfection

Human glioma cell lines, LN118, SHG44, U87, U251, and A172, were obtained from Cell Bank of Chinese Academy of Sciences (Pudong, Shanghai, China). The cells were grown in the Roswell Park Memorial Institute (RPMI) 1640 medium, supplemented with 10% fetal bovine serum (FBS) (Yunshan Technology, Haidian, Beijing, China), 100 U/ml penicillin G (Yunshan Technology, Haidian, Beijing, China), and 100 *μ*g/ml streptomycin (Sigma, Suzhou, Jiangsu, China). Normal human astrocytes (NHA) were obtained from Weihui Biot (Hangzhou, Zhejiang, China) and were grown in Dulbecco's Modified Eagle's Medium with high glucose and sodium pyruvate.

The siRNAs used to knockdown ZNF667-AS1 were purchased from GEMA GENE (Pudong, Shanghai, China). The siRNA sequences were as follows: si-ZNF667-AS1: CTACCACAGCTTCCATG; si-ZNF667-AS1-2: GCCCACTGTATTCAACA. Cell transfection was conducted by using the LipofectamineTM 2000 transfection reagent (Invitrogen, Guangzhou, Guandong, China) according to the manufacturer's instructions. After transfection for 24 h, the knockdown efficiency was evaluated by RT-PCR.

### 2.3. Reverse Transcription and qPCR Analyses

Total RNA from glioma specimens and nontumor specimens was extracted with the Trizol reagent (Takara, Kunshan, Jiangsu, China) by complying with the plan of the producer. RNA concentration was ascertained; this study adopted 100 *μ*g of RNA during reverse transcription. Based on an ABI 7900 Real-Time PCR System, this study conducted qPCR with the plan below: 95°C for 3 min., 40 cycles of 95°C for 15 sec., 60°C for 15 sec., and 72°C for 30 sec. The comparative expressing level of ZNF667-AS1 underwent the normalization to GAPDH. The primers included ZNF667-AS1 forward, 5′-GGTCCACTTCACGCACTTGC-3′; ZNF667-AS1 reverse, 5′-ACCATTCGAACTTGGCTACA-3′; GAPDH forward, 5′-GTCAACGGATTTGGTCTGTA-3′; GAPDH reverse, 5′-AGTCTTCTGGGTGGCAGTG-3′. Using the 2^-*ΔΔ*Ct^ approach, the relative expression conditions of the gene of interest were obtained. qRT-PCR reaction processes were overall performed 3 times.

### 2.4. Cell Proliferation Assays

For the cell proliferation analysis, 300 living cells (A172 and LN118 cells) were transfected with either si-ZNF667-AS1-1, ZNF667-AS1-2, or a scrambled control and plated onto 96-well plates. Every 24 h, the CCK-8 solution (10 *μ*l) was added to three randomly selected wells, and the cultures were incubated at 37°C for 90 min. The absorbance was measured at 450 nm using a microplate reader.

### 2.5. Statistical Analysis

Statistical analysis was conducted with SPSS version 13.0 software (SPSS Inc, Chicago, IL, USA). With the Student's *t*-test, the diversifications of two cohorts were discussed. The relationship between the ZNF667-AS1-expressing level and clinical-pathological characteristics was analyzed with the chi-square test. Based on the Kaplan-Meier approach, the overall survival (OS) and disease-free survival (DFS) ratios were obtained with the log-rank experiment adopted to draw the comparing process. Multivariate Cox regression analyses were carried out for analyzing the survival information. A *p* value <0.05 was suggested to exhibit statistical significance.

## 3. Results

### 3.1. ZNF667-AS1 Was Upregulated in Glioma

**Figure 2:**
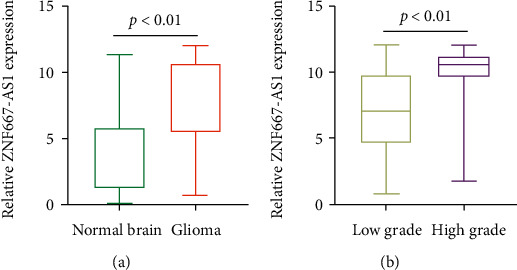
The ZNF667-AS1 expression is increased in glioma tissues. (a) The relative expression of ZNF667-AS1 in paired tumor and nontumor tissues (*n* = 155) by RT-PCR. The expression of ZNF667-AS1 in different tumor grades.

To determine the possible functions of ZNF667-AS1 in glioma, we enrolled 155 glioma patients and performed RT-PCR to examine the expression of ZNF667-AS1 in a total of 155 paired glioma tissues and corresponding nontumor tissues. As shown in Figure 2(a), we observed that the ZNF667-AS1 expression was distinctly increased in glioma specimens compared to matched nontumor specimens (*p* < 0.01). Besides, we also observed that glioma specimens with advanced stages exhibited a higher level of ZNF667-AS1 than those with early stages (*p* < 0.01, Figure 2(b)). Overall, our findings suggested ZNF667-AS1 as a functional regulator in glioma progression.

### 3.2. The Diagnostic Significance of the Overexpression of ZNF667-AS1 in Glioma

**Figure 3:**
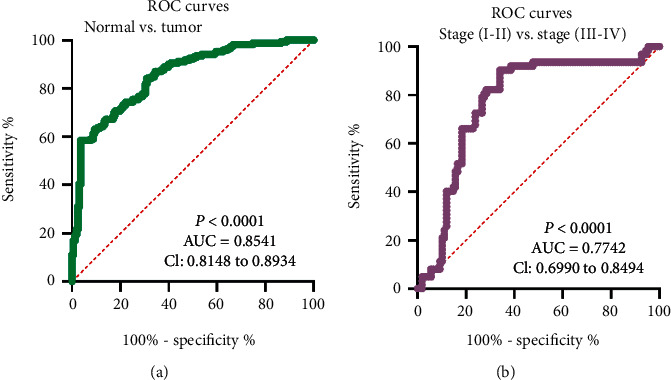
ZNF667-AS1 may be a potential diagnostic biomarker for glioma patients. (a) The ROC curve for the diagnostic value of ZNF667-AS1 in distinguishing glioma specimens from nontumor brain specimens. (b) The ROC curve for the diagnostic value of ZNF667-AS1 in distinguishing glioma specimens with stages (I-II) from glioma specimens with stages (III-IV).

Previous studies have revealed several functional lncRNA that displayed a diagnostic value in glioma patients. Then, we performed ROC assays which showed that the high ZNF667-AS1 expression had an AUC value of 0.8541 (95% CI: 0.8148 to 0.8934) for glioma (Figure 3(a)). The sensitivity and specificity of ZNF667-AS1 expressions for distinguishing glioma samples from normal samples were 68.22%/84.57%. In addition, we also observed that ZNF667-AS1 could be used as a molecular marker for distinguishing glioma specimens with advanced stages from glioma specimens with early stages with an AUC of 0.7742 (95% CI: 0.6990-0.8494; *p* < 0.01, Figure 3(b)). Our findings suggested ZNF667-AS1 as an early diagnosis indicator for glioma patients.

### 3.3. Correlations between ZNF667-AS1 and Clinical Features of Glioma

For better understanding of the clinical relevance of the ZNF667-AS1 expression in glioma, the 155 glioma patients were split as a high expression cohort (*n* = 79) and a low expression cohort (*n* = 76), complying with the median expressing condition of ZNF667-AS1 (5.62) in all glioma samples. Then, our group determined the possible association between ZNF667-AS1 levels and several clinical factors using the chi-square test which suggested that the high ZNF667-AS1 expression was associated with the WHO grade (*p* = 0.005) and KPS score (*p* = 0.008, Table 1). However, there were no significant correlations of the ZNF667-AS1 expression with other clinical features (*p* > 0.05).

### 3.4. The Possible Prognostic Values of the ZNF667-AS1 Expression in Glioma

**Figure 4:**
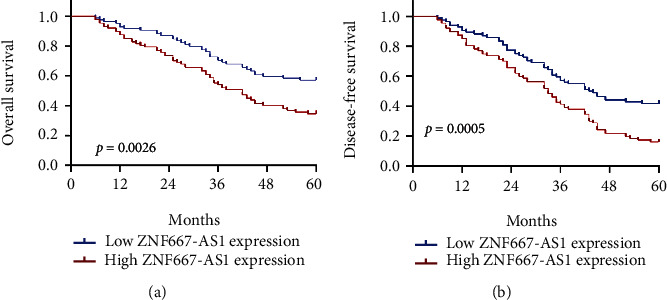
Kaplan-Meier curves of the overall survival (a) and disease-free survival (b) of 155 glioma patients.

To further explore whether ZNF667-AS1 may display a positive influence on the survivals of glioma patients, we collected the survival data of all 141 patients who completed five-year follow-up, which were analyzed using the Kaplan-Meier survival curves. Interestingly, our results revealed that patients with the high expression of ZNF667-AS1 had shorter OS (*p* < 0.0026; Figure 4(a)) and DFS (*p* < 0.005; Figure 4(b)) as compared with the ZNF667-AS1 low group. More importantly, in a multivariate Cox model, we demonstrated that the ZNF667-AS1 expression was an independent poor prognostic factor for both 5-year OS (HR = 2.897, 95% CI: 1.365-4.784, *p* = 0.008) and 5-year DFS (HR = 3.019, CI =1.414-4.899, *p* = 0.005) in glioma (Table 2).

### 3.5. Effects of ZNF667-AS1 on Proliferation of Glioma Cells

**Figure 5:**
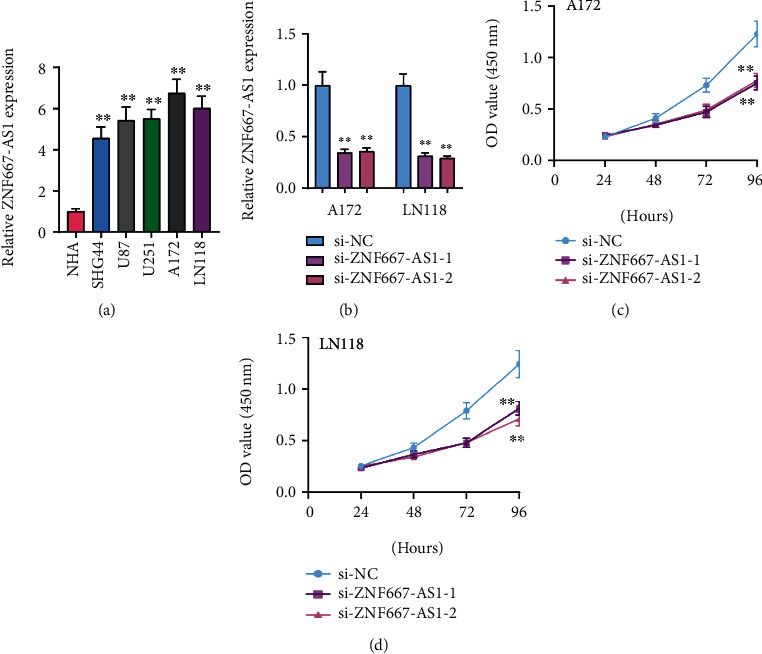
Knockdown of ZNF667-AS1 silencing inhibits the proliferation of A172 and LN118 cells. (a) RT-PCR for the demonstration of the ZNF667-AS1 expression in five glioma cells and NHA. (b) qPCR analyses of ZNF667-AS1 levels following treatment of A172 and LN118 cells with si-ZNF667-AS1-1, ZNF667-AS1-2, or si-NC. (c, d) The MTT assay was performed to determine the proliferation of A172 and LN118. ^∗∗^*p* < 0.05.

To analyze the possible function of ZNF667-AS1 on the proliferation of glioma cell, we firstly examine the expression of ZNF667-AS1 in five glioma cells, finding that ZNF667-AS1 was highly expressed in five glioma cells compared to NHA (Figure 5(a)). Because A172 and LN118 cells exhibited a higher level of ZNF667-AS1, we chose them for the following experiments. Real-time PCR was performed to confirm the successful knockdown of ZNF667-AS1 in A172 and LN118 cells after the transfection of si-ZNF667-AS1-1 and si-ZNF667-AS1-2 (Figure 5(b)). CCK-8 assays revealed that knockdown of ZNF667-AS1 significantly suppressed cell proliferation in A172 and LN118 cells (Figures 5(c) and 5(d)). Overall, our findings suggested that ZNF667-AS1 served as a tumor promotor in glioma.

## 4. Discussion

Glioma is the most common intracranial malignant tumor in humans [1, 24]. Highly glioma presents unique challenges due to its tendency to proliferate and invade tissues. Malignant gliomas, including glioblastomas, with radiotherapy and temozolomide have only a small survival benefit [25, 26]. The sensitive biomarkers were very important for the treatments of tumor patients [27, 28]. However, none of the currently identified biomarkers are sensitive or specific enough for reliable glioma screening in clinical settings. In recent years, growing studies have indicated that some functional lncRNAs could be used as novel biomarkers for prognosis, survival, and responses to the treating process that paved a novel wave of research into molecular markers of glioma [29, 30]. In this study, we forced on a novel glioma-related lncRNA ZNF667-AS1.

In recent years, several studies have reported the dysregulated expression of ZNF667-AS1 and its possible effects in several tumors. For instance, Chen et al. [21] reported that the expression of ZNF667-AS1 was distinctly decreased in nasopharyngeal carcinoma specimens and cell lines. Functionally, the overexpression of ZNF667-AS1 was shown to suppress the proliferation of nasopharyngeal carcinoma cells via increasing the ABLIM1 expression via adsorbing miR-1290. Li et al. [23] showed that ZNF667-AS1 was significantly highly expressed in cervical cancer, and its low levels suggested a poor prognosis of cervical cancer patients. In vitro and in vivo assays indicated that the forced expression of ZNF667-AS1 inhibited the proliferation and metastasis through counteracting the miR-93-3p-dependent PEG3 suppression. Moreover, ZNF667-AS1 was also shown to suppress the inflammatory response, promoting the recovery of spinal cord injury via suppressing the JAK-STAT pathway [31]. The negatively regulatory association between ZNF667-AS1 and JAK-STAT pathway highlighted its potential effects acting as tumor suppressors in tumors due to the important effects of the JAK-STAT pathway in tumor progression. All previous findings suggested ZNF667-AS1 may be a critical regulator in several tumor kinds. However, the expressing and clinical value of ZNF667-AS1 in glioma remained unclear.

Here, the expressing condition of ZNF667-AS1 in 155 glioma patients was first examined, confirming that the ZNF667-AS1 expression was distinctly upregulated in glioma specimens in comparison with matched nontumor brain tissues, which was inconsistent with its expression trend in other several tumors. Then, we determined the diagnostic value in glioma patients and found that ZNF667-AS1 in tissues effectively differentiated glioma tissues from nontumor specimens with an area under the ROC curves (AUC) of 0.8541. The diagnostic value of the ZNF667-AS1 expression was also confirmed in patients with different stages. Then, we analyzed the clinical significance of the ZNF667-AS1 expression, finding that the high ZNF667-AS1 expression was associated with the KPS score and advanced WHO grade, suggesting it that acted as a tumor promotor in clinical progression of glioma. Moreover, after analyzing the survival data with five-year following-up, we confirmed that patients with high ZNF667-AS1 expressions exhibited a longer OS and DFS than those with low ZNF667-AS1 expressions. Finally, the results of the multivariate study verified ZNF667-AS1 a single predictor for OS and DFS in glioma patients. On the other hand, we also performed CCK-8 assays to explore the tumor-related effects of ZNF667-AS1 on glioma cells, confirming that knockdown of ZNF667-AS1 suppressed the proliferation of glioma cells. Our findings suggested ZNF667-AS1 as a tumor promotor in glioma progression.

Some limitations of this study should be noted. First, because of the small number of patients analyzed in our research, more patients for clinical experiments are required to confirm our findings. Second, we fail to determine whether the serum ZNF667-AS1 expression was dysregulated in glioma patients. The serum biomarkers are very important for the monitor of the therapy response in real-time. Third, the potential effects of ZNF667-AS1 on the metastasis ability and in vivo assays are not performed. In the future, we plan to more fully describe the mechanisms and relationships with lncRNAs related to glioma biomarkers.

## 5. Conclusions

The results revealed initially that ZNF667-AS1 may represent a valuable independent prognostic indicator for glioma. Further study to discover the molecular mechanism of ZNF667-AS1 about tumor migration and invasion is currently in progress.

## Figures and Tables

**Table 1:** The association between the ZNF667-AS1 expression and different clinicopathological features of 155 human gliomas.

Parameter	No. of cases	ZNF667-AS1 expression		*p* value
		High	Low	
Age				0.369
<50	82	39	43	
≥50	73	40	33	
Gender				0.394
Male	93	50	43	
Female	62	29	33	
WHO grade				0.005
I-II	108	47	61	
III-IV	47	32	15	
KPS score				0.008
<80	57	37	20	
≥80	98	42	56	
Tumor size				0.145
<3 cm	95	44	51	
≥3 cm	60	35	25	

**Table 2:** Multivariate analysis of the overall survival and disease-free survival in glioma patients.

Variables	Overall survival			Disease free survival		
	HR	95% CI	*p* value	HR	95% CI	*p* value
Age	0.882	0.452-1.342	0.233	1.113	0.673-1.732	0.143
Gender	1.442	0.642-2.104	0.342	1.632	0.773-2.341	0.219
WHO grade	2.893	1.324-4.872	0.009	3.014	1.432-5.118	0.005
KPS score	2.782	1.423-4.563	0.013	2.964	1.395-4.895	0.007
Tumor size	1.237	0.672-1.897	0.213	1.427	0.875-2.137	0.136
ZNF667-AS1 expression	2.897	1.365-4.784	0.008	3.019	1.414-4.899	0.005

## Data Availability

The data used to support the findings of this study are available from the corresponding author upon request.
